# QRREM method for the isolation of high-quality RNA from the complex matrices of coconut

**DOI:** 10.1042/BSR20181163

**Published:** 2019-01-03

**Authors:** Amjad Iqbal, Yaodong Yang, Rashad Qadri, Yi Wu, Jing Li, Farooq Shah, Muhammad Hamayun, Anwar Hussain

**Affiliations:** 1 Hainan Key Laboratory of Tropical Oil Crops Biology/Coconut Research Institute, Chinese Academy of Tropical Agricultural Science, Wenchang, Hainan 571339, China; 2 Department of Agriculture, Abdul Wali Khan University Mardan, 23200 Pakistan; 3 Institute of Horticultural Sciences, University of Agriculture Faisalabad, 38000 Pakistan; 4 Department of Botany, Abdul Wali Khan University Mardan, 23200 Pakistan

**Keywords:** complex matrices, cDNA library, coconut, betel nut, RNA extraction

## Abstract

Complex plant tissues vary in hardness, i.e. some are succulent, while others are complex to break. Besides, plant metabolites, such as polysaccharides, proteins, polyphenols and lipids, can greatly interfere with the RNA extraction. So, in order to obtain a high-quality RNA from the complex tissues (like coconut endosperm, coconut apple and coconut leaf bud) rich in secondary metabolites, a robust method is demanded. Several methods (MRIP, CTAB and TRIZOL) have been used previously for the isolation of quality RNA from the coconut tissues, but without any success. The present study will provide with the details of a new method (Quick and Reliable RNA Extraction Method or QRREM), which have efficiently isolated the intact RNA form the complex tissues of coconut compared with CTAB, Trizol and RNA plant. The method has been validated for the isolation of high-quality intact RNA from the other available plant species (Areca/betel nut, mint and spring onion). The method has various advantages over the other methods in terms of time and cost effectiveness. Furthermore, the resulted RNA from various tissues of coconut performed well in the downstream experiments, i.e. reverse transcription and PCR for the production and amplification of cDNA.

## Introduction

Many plant species contains complex tissues and higher concentrations of plant metabolites, which can interfere with the extraction of RNA. The co-precipitation of these metabolites restricts the chances of getting high-quality intact RNA [1–3]. Among the secondary metabolites, alkaloids and polyphenols are well known for their affinity to RNA. Alkaloids and polyphenols can form a complex network with RNA, causing difficulties in isolation [4,5]. However, for the construction of complementary DNA (cDNA) library, RNA sequencing, reverse transcription real-time PCR (RT-PCR), quantitative real-time PCR (RT-qPCR) and gene analysis, an intact RNA with high purity is essential [6–9]. Therefore, it is extremely vital to introduce a new method that can remove all the interfering components and isolate an intact RNA in high quantities that can be used in downstream experiments.

Recently, a number of methodologies and commercial kits have been developed to extract high-quality RNA from various complex matrices. The commercial kits from Invitrogen TRIZOL Reagents are available for the isolation of RNA from the starch rich tissues of rice [10], oil rich tissues of rapeseed [11] and Arabidopsis [12]. But the method was unsuccessful in extracting RNA from the palm species [7] that are rich in polysaccharides and polyphenols. Tiangen has also introduced their own kit (Tiangen RNAplant reagent kit) for better isolation of RNA from potato tubers, banana, apple and pear tissues that are rich in starch [13]. Also, lithium chloride has been proposed for the extraction of RNA from polyphenols rich tissues [14]. Conversely, the method used for the isolation of RNA from complex plant tissues that got great attention in recent years is cetyltrimethylammonium bromide (CTAB) [2]. No doubt, the method bears a low operational cost, yet time-consuming, which can cause the degradation of RNA during the extensive isolation procedure. Additionally, the co-isolation of DNA is very common that demands additional steps to purify RNA, resulting in a possible degradation of RNA. Similarly, Xioa et al. [7] has also developed MRIP method (Method for RNA isolation from Palm) that revealed promising results regarding the leave tissues of palm, though lacking precision and accuracy for other tissues. Guanidinium-phenol-chloroform, on the other hand, is one of the promising methods for the isolation of RNA from animal tissues, while it has shown low success with plant tissues [9].

In the present study, a new method (QRREM) that gave consistent results is discussed; the method is robust, less time consuming (taking up to 1.5 h, including qualitative and quantitative analysis), cheap and reliable to isolate RNA from the very complex matrices of coconut tissues (rich in polysaccharides, lipids, proteins and polyphenols). The method has provided with an intact RNA in high quantities, which were actively utilized in downstream experiments. This method has primarily been developed for the isolation of RNA from the coconut tissues, but exhibited promising results for the other plant species and would be worth trying on animal tissues.

## Materials

Agrose gel, chloroform, chloroform-isoamyl alcohol (24:1, i.e. 24 parts of chloroform mix with 1 part of isoamyl alcohol), acid buffered phenol (pH 4.5), isopropanol, ammonium thiocynate, guanidine thiocynate, sodium acetate, glacial acetic acid, glycerol, β-merceptoethanol, polyvinylpyrrolidone (PVP)-40, RNase free tubes, mortar and pestle, scissor/razor blade, plastic bags, freezer, spatula, pipette, Tris borate EDTA (TBE) buffer, ethidium bromide, DNA ladder and diethylpyrocarbonate (DPEC)-treated water. All the chemicals used were of high analytical purity and were purchased from Real Times and Chemical Reagents, China. TaKaRa, PrimeScript II 1^st^ strand cDNA synthesis kit was used for reverse transcription and PCR kit from Real Time was used for the amplification of cDNA.

## Equipment

Agrose gel electrophoresis system

Bench centrifuge (Eppendorf Centrifuge 5804 R)

Vortex (Vortex-BE1, Kylin-Bell Lab Instruments)

Gel Doc system (Syngene G: Box F3, Gene Company Limited)

Nanodrop (Nanodrop 2000 spectrophotometer, Thermo Scientific)

PCR machine/thermocycler (TaKaRa thermocycler dice touch TP350, TaKaRa Bio Incharge)

## Plant materials

The coconut tissues of freshly harvested coconuts were collected at the Coconut Research Institute, Chinese Academy of Tropical Agricultural Sciences, Wenchang, Hainan-China. Similarly, leaf tissues of betel nut, spring onion and mint were taken from the freshly sown vegetables at the vegetable farm of the same Institute. All the samples were cut into small pieces with the help of scissors or razor blade and transferred to small plastic bags. The plastic bags were then preserved quickly in liquid nitrogen and were kept frozen at −80°C till further analysis.

## Buffer recipe

- Ammonium thiocyanate: 0.4 M
- Guanidine thiocyanate: 0.7 M
- 3 M sodium acetate (pH 5): 3.3 ml
- Phenol: 38 ml
- Glycerol: 5 ml
- DEPC-treated water: up to 100 ml
- β-Merceptoethanol: 2%
- PVP-40: 3%

## Preparation

Take 30 ml of (DEPC-treated water) and add the contents to it one by one (except PVP-40), once dissolved the volume should be made up to 100 ml with DPEC-treated water. The buffer should be handled in a fume hood as it contains phenol and should be kept refrigerated in a dark bottle to avoid oxidation. The buffer can be used within 6 months of preparation.

### Critical step

If PVP-40 is added to the buffer it will react with ammonium thiocynate, giving a pink color, which tends to decrease the performance of the extraction buffer significantly. Therefore, PVP-40 should be added to the mortar before crushing the sample in liquid nitrogen.

## QRREM protocol

**Figure 1:**
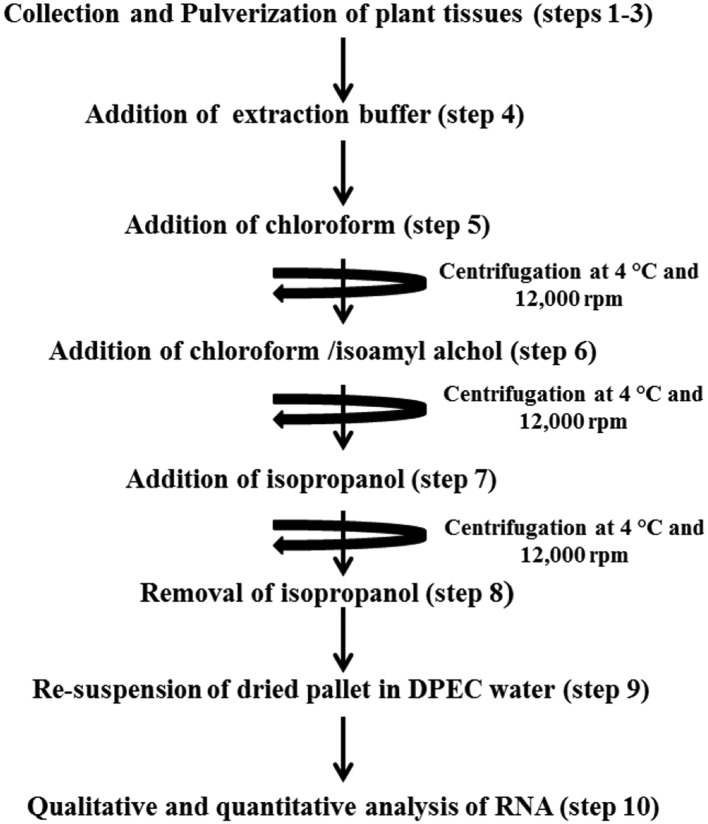
Flowchart representing the concise steps in QRREM method The detail of each step is given in protocol section.

All the steps in the protocol are summarized in a flowchart (Figure 1).

## Tissue collection and pulverization

- Transfer the sample containing bag from the freezer into liquid nitrogen to avoid any thawing before the homogenization of the tissue.
- Pour liquid nitrogen into a clean mortar and pestle (previously autoclaved) to make it cool.
- Take approximately 0.08 g of the frozen tissue from the plastic bag and transfer it to the mortar containing liquid nitrogen and PVP-40 (3%). Grind the tissue to a fine powder with a pestle, top up the mortar with liquid nitrogen during grinding to avoid any thawing.

## Extraction of RNA

(iv) Immediately transfer pulverized tissue with the help of a frozen spatula to a 1.5-ml tube, containing 1 ml of extraction buffer, vortex for 15 s, and then incubate on ice for 5 min.
(v) Add 200 µl of chloroform and vortex thoroughly for 15 s, then incubate on ice for 10 min. Centrifuge it for 5 min at 12000×***g*** at 4°C.
(vi) Transfer the supernatant (500 µl) to a new RNase free tube and add an equal volume of chloroform/isoamyl alcohol to it and centrifuged at 12000×***g*** for 5 min.

## Precipitation of RNA

(vii) Carefully transfer the upper aqueous phase (400 µl) of the solution to a fresh, RNase-free tube, then add an equal volume of ice cold isopropanol (500 or 600 µl), mix the solution by inverting the tubes and incubate for 5 min on ice.
(viii) Centrifuge for 5 min at 12000×***g*** and 4°C, after centrifugation discard the supernatant. The RNA pellet is generally at the bottom of the tube. Remove all the residual isopropanol by placing the tube upside down and let the pellet dry for 5 min.
(ix) Redissolve RNA with 30 μl of RNase-free (DEPC-treated) water by pipetting gently for 1 to 2 min.

## Qualitative and quantitative analysis of RNA

(x) The quantity and purity of the RNA were checked on a Nanodrop, while the quality was confirmed by running an agrose gel electrophoresis.
  - Nanodrop 2000 analysis: The concentration of RNA was determined spectrophotometrically by applying 1 μl of sample to the Nanodrop 2000 and measured the absorbance at 260 and 280 nm. The absorbance at *A*_260_ had computed the quantity of the RNA, where the purity of RNA was checked by *A*_260/280_ ratio, which is in the range from 1.8 to 2.2.
  - Agrose gel electrophoresis: We prepared a 0.8% (w/v) agrose gel by dissolving 0.2 g of RNA grade agrose in 25 ml of 0.5× TBE buffer in an RNase-free conical flask. The flask was heated in a microwave oven till the complete dissolution of agrose in the TBE buffer. Ethidium bromide (2 μl) was added to the content, and the flask was swirled. The mixed content of the flask was then poured into the gel tray with a comb and was left to set for approximately 20 min. After setting of the gel, RNA loading buffer (2 μl) was added to the sample (3 μl) and loaded onto the gel. The gel was run for 12 min at 120 V and 240 mA.

## Break up of time

Pulverization of sample: steps (i) to (iii) took 20 min for four samples

RNA extraction: steps (v) to (vi) took 15 min

RNA precipitation: steps (vii) to (iv) took 20 min

Qualitative and quantitative analysis of RNA: step (x) (a and b) took 25 min

## Reverse transcription or cDNA synthesis

The first strand cDNA was synthesized by using TaKaRa PrimeScript II 1^st^ strand cDNA synthesis kit. According to the manufacturer’s protocol, the reaction of the RT was conducted as follows: approximately 2 μg of the isolated RNA was mixed with 1 μl oligo dT primer (50 μM) + dNTP mixture (10 mM) and made the volume to 10 μl with DPEC water in a Microtube. The tube was incubated at 65°C for 5 min, and then chilled on ice. In the next step, 5x PrimeScript II buffer (4 μl), RNase inhibitor (0.5 μl) and PrimeScript II RTase (1 μl) were added to the tube and the final volume was brought up to 20 μl with DPEC water. The mixture was incubated at 42°C for 60 min. To inactivate the enzymes, the mixture was incubated for further 5 min at 95°C, followed by cooling on ice.

## RT-PCR

After the synthesis of first strand cDNA, RT-PCR was done using a kit from the Real Times to amplify the cDNA. An LPAAT-α gene was tested using primers: CnLPAAT-For: ATGGATGCTTCAGGGGCAAGTTCG and CnLPAAT-Re: TTATGAATTTGACCTTCCGCTAGCATCC for coconut endosperm and coconut apple samples, whereas a housekeeping gene Actin were tested using primers; CnACT-For: ATAAAGTATGGCTGATGCTGAGG and CnACT-Re: CAACAATGCTTGGGAACACA for coconut leaf bud. cDNAs were amplified by mixing 2 μl of cDNA, 1 μl of reverse primer (10 μM), 1 μl of forward primer (10 μM), 5 μl 10x TAQ buffer, 1 μl dNTP mix (10 mM), 0.5 μl TAQ (5U/μl) and 39.5 μl ddH_2_O in a Microtube. The tube was then placed in a thermocycler with the following settings: 94°C for 3 min; 35 cycles (94°C for 30 s, 54°C for 30 s, 72°C for 1 min); 72°C for 5 min.

## Results

**Figure 2:**
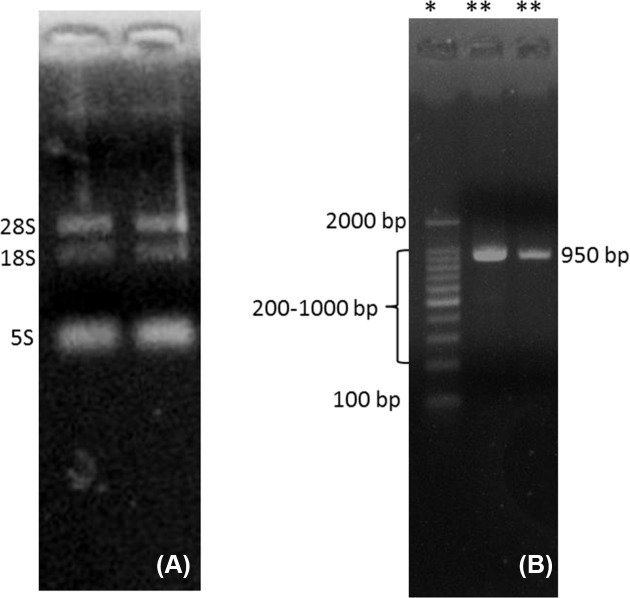
Isolation of RNA from coconut endosperm by QRREM method (**A**) represents RNA bands that have been obtained after running 3 µl of extracted RNA from the fresh coconut endosperm by QRREM method on the agrose gel for 12 min; 28S, 18S and 5S represent the different bands of rRNA. Lane 1 of (**B**) with ‘*’ sign represents the marker (DNA ladder), whereas lane 2 and 3 with ‘**’ represents the samples. Lanes 2 and 3 of (B) represents the RT-PCR product of RNA isolated from fresh coconut endosperm by QRREM method. Each lane of the agrose gel in (B) was loaded with 3 µl of DNA marker or RT-PCR product. The gel was run for 20 min and the bands were observed in the Gel Doc system.

**Figure 3:**
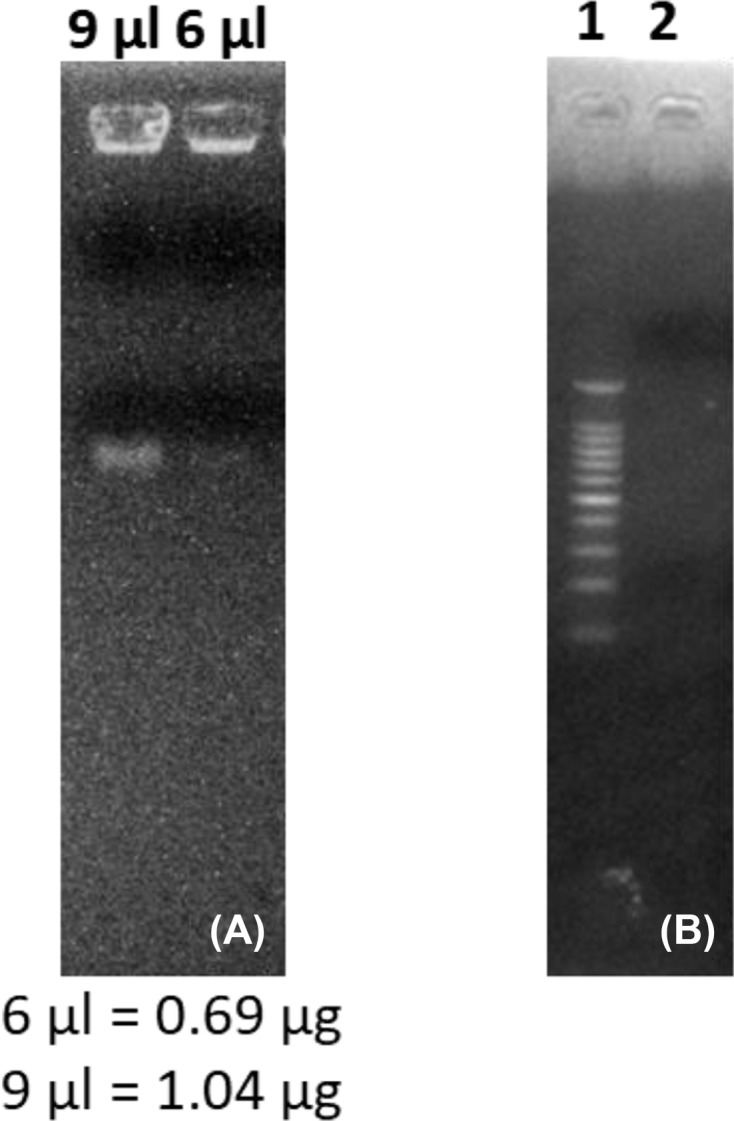
Isolation of RNA from coconut endosperm by Trizol method (**A**) represents the degraded RNA bands that have been obtained after running 6 and 9 µl of extracted RNA from the fresh coconut endosperm by Trizol method on the agrose gel for 12 min; lane 1 of (**B**) represents the marker (DNA ladder), whereas lane 2 represents the samples. Lane 2 of (B) represents the RT-PCR product of RNA isolated from fresh coconut endosperm by Trizol method. Each lane of the agrose gel in (B) was loaded with 3 µl of DNA marker or RT-PCR product. The gel was run for 20 min and the bands were observed in the Gel Doc system.

**Figure 4:**
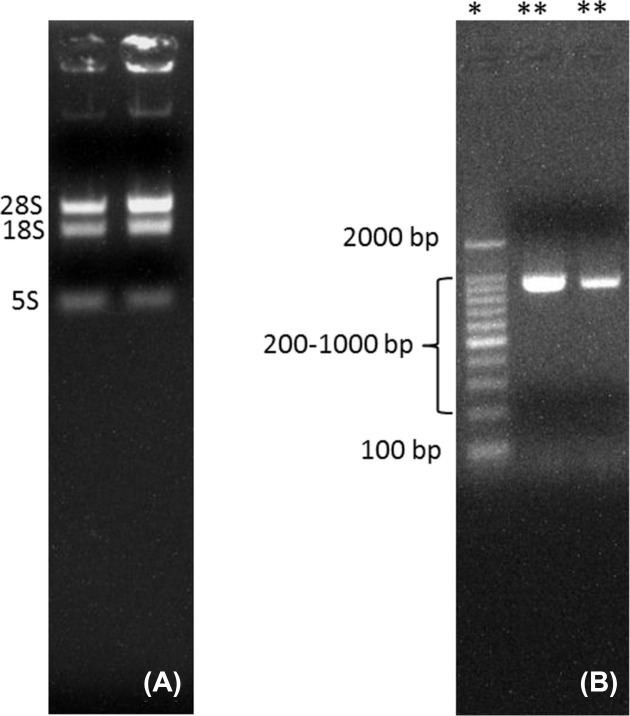
Isolation of RNA from coconut apple by QRREM method (**A**) represents RNA bands that have been obtained after running 3 µl of extracted RNA from the coconut apple by QRREM method on the agrose gel for 12 min; 28S, 18S and 5S represent the different bands of rRNA. Lane 1 of (**B**) with ‘*’ sign represents the marker (DNA ladder) of different molecular weight, whereas lanes 2 and 3 with ‘**’ represent the samples. Lane 2 of (B) represents the RT-PCR product of RNA isolated from the coconut apple by QRREM method. Each lane of the agrose gel in (B) was loaded with 3 µl of DNA marker or RT-PCR product. The gel was run for 20 min, and the bands were observed in the Gel Doc system.

**Figure 5:**
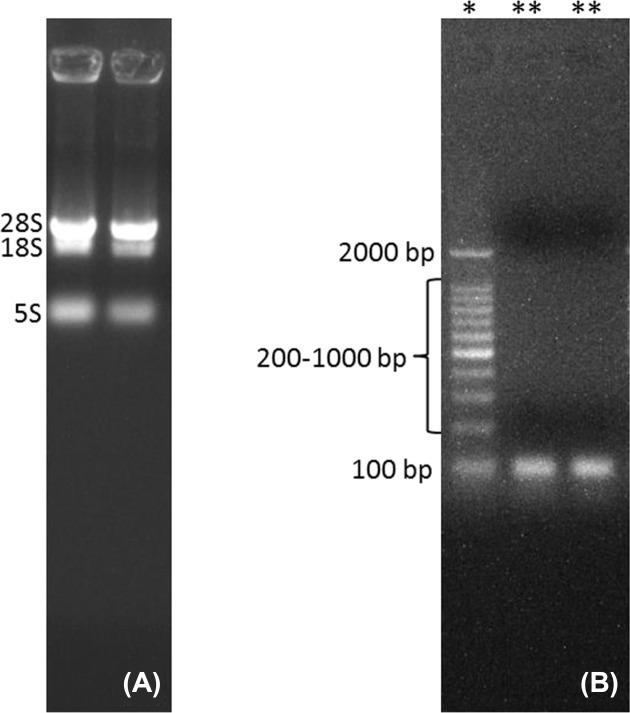
Isolation of RNA from coconut leaf bud by QRREM method (**A**) represents RNA bands that have been obtained after running 3 µl of extracted RNA from the coconut leaf bud by QRREM method on the agrose gel for 12 min; 28S, 18S and 5S represent the different bands of rRNA. Lane 1 of (**B**) with ‘*’ sign represents the marker (DNA ladder) of different molecular weight, whereas lanes 2 and 3 with ‘**’ represent the samples. Lane 2 of (B) represents the RT-PCR product of RNA isolated from the coconut leaf bud by QREEM method. Each lane of the agrose gel in (B) was loaded with 3 µl of DNA marker or RT-PCR product. The gel was run for 20 min, and the bands were observed in the Gel Doc system.

**Figure 6:**
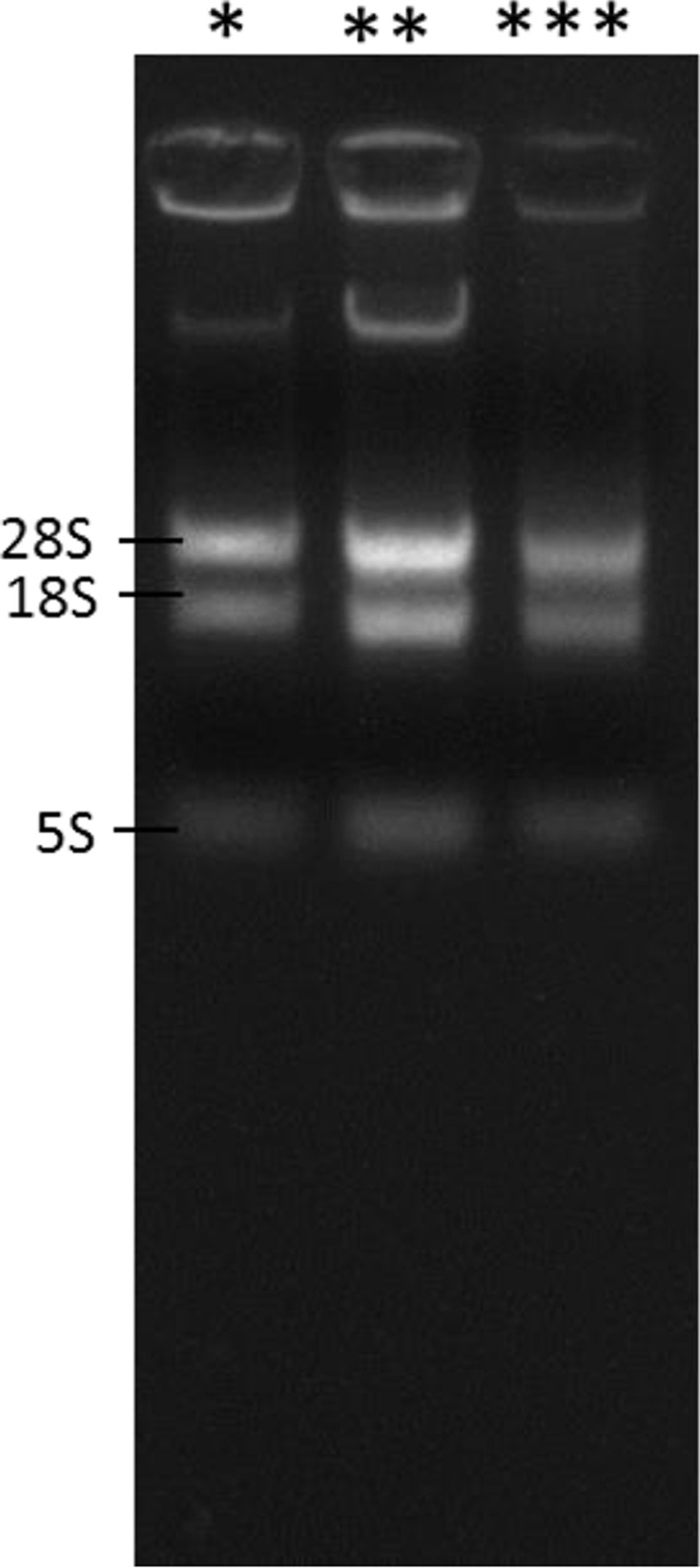
Validation of the QRREM method by isolating RNA from available plant tissues This figure represents RNA bands that have been obtained after running 3 µl of extracted RNA from available plant species by QRREM method on the agrose gel; 28S, 18S and 5S represent the different bands of rRNA. ‘*’ represents the extracted RNA from betel nut, ‘**’ represents the extracted RNA from mint and ‘***’ represents the extracted RNA from spring Onion. The gel was run for 12 min, and the bands were observed in the Gel Doc system.

**Figure 7:**
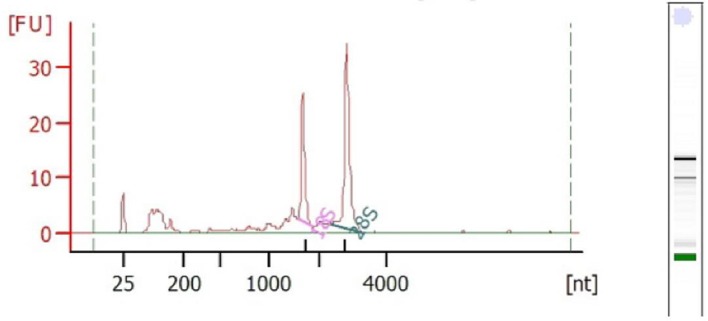
Analysis of RNA isolated by QRREM method with Agilent 2100 Bioanalyzer microfluidic electrophoresis chip The electropherogram of the RNA samples extracted with QRREM method helps to determine the intensity of each band on the gel. The arrows indicate 28S and 18S ribosomal bands (FU = Fluorescence Units, nt = nucleotide). The RIN of the extracted RNA = 9.1 and the 28S/18S ratio = 1.8.

The newly developed protocol for the extraction of RNA from complex matrices, rich in carbohydrates, lipids, proteins and polyphenols provided us with a high-quality RNA in appreciable quantities when compared with the CTAB, Trizol and RNAplant methods (Table 1). The *A*_260/280_ and *A*_260/230_ ratios of the extracted RNA by the QRREM method were ranged from 1.90 to 2.00 and 1.97 to 2.01 for various tissues of coconut. Moreover, *A*_260/280_ and *A*_260/23_0 ratios of the extracted RNA by the QRREM method were ranged from 1.93 to 2.03 and 1.98 to 2.00 for betel nut, mint and spring onion. Similarly, the concentration of the extracted RNA from various tissues of coconut by the QRREM method was in the range from 16.2 to 63.2 µg/80 mg tissue, where for other plant species (betel nut, mint and spring onion) the values were between 12.7 and 20.9 (Table 2). On the contrary, CTAB and RNA plant methods yielded very low amounts of impure RNA (Table 1) that were not enough to perform electrophoresis. The RNA obtained by Trizol method was higher (i.e. 10.4 ± 1.53 µg/ 80 mg tissue) as compared with the CTAB and RNA plant methods, but the quality was very poor (Table 1). From the electrophoretogram, it was quite clear that RNA extracted from the coconut endosperm by QRREM method consisted of 28S, 18S and 5S rRNA bands without any contamination (Figure 2A). Conversely, the RNA extracted from the coconut endosperm by Trizol method consisted of one band even at higher loading concentrations (Figure 3A). That means that the quality of RNA extracted by Trizol method was very poor. Furthermore, the 28S rRNA band of the extracted RNA from various tissues of coconut and other species by QRREM method was far more intense than the 18S rRNA band that indicates the high quality of extracted RNA (Figures 4–6). The quality of RNA was further checked with the synthesis and amplification of the cDNA by RT-PCR. The results indicated that RNA that has been extracted from the coconut tissues by the QRREM method that has successfully produced the amplified RT-PCR product (Figures 2B, 4B and 5B). Whereas the RNA obtained by the Trizol method was failed to produce cDNA (Figure 3B). The quality of RNA isolated with the QRREM was further assessed by an Agilent 2100 Bioanalyzer microfluidic electrophoresis chip, and the results were consistent with the agarose gel electrophoresis analysis of the RNA (Figure 6). RNA isolated with the QRREM protocol exhibited three peaks, with the highest peak corresponding to the 28S rRNA, an rRNA 28s/18s ratio of 1.8 and a RIN (RNA integrity number) value of 9.1, indicating that the mRNAs were of good integrity. The gel electrophoresis and the microfluidic electrophoresis (Figure 7) equally indicated that the QRREM protocol is suitable for isolating high-quality RNA from coconut tissues.

**Table 1:** Concentration and quality of RNA from coconut endosperm by QRREM, CTAB, Trizol and RNA plant methods

Coconut endosperm	Conc. (µg/80 mg)	*A*_260/280_ ratio	*A*_260/230_ ratio
QRREM	16.2 ± 0.11	1.90 ± 0.01	1.97 ± 0.05
CTAB	0.1 ± 0.03	0.64 ± 0.11	1.81 ± 0.07
Trizol	10.4 ± 1.53	1.38 ± 0.03	1.70 ± 0.09
RNAplant	0.5 ± 0.15	1.83 ± 0.01	1.90 ± 0.04

A 1 µl drop was loaded onto the Nanodrop 2000.

**Table 2:** Concentration and quality of RNA from various tissues of coconut and other species by QRREM method

Sample tissue	Conc. (µg/80 mg)	*A*_260/280_ ratio	*A*_260/230_ ratio
Coconut apple	18.7 ± 0.74	1.97 ± 0.06	1.99 ± 0.08
Coconut leaf bud	63.2 ± 0.52	2.00 ± 0.01	2.01 ± 0.06
Betel nut leaf	17.9 ± 1.26	1.93 ± 0.02	1.98 ± 0.04
Mint leaf	20.9 ± 0.04	2.03 ± 0.05	2.00 ± 0.09
Spring onion	12.7 ± 1.57	1.96 ± 0.01	1.99 ± 0.03

A 1 µl drop was loaded onto the Nanodrop 2000.

RNA extraction is the initial and most important step for various investigations in the field of molecular biology, including RNA sequencing, cDNA library construction and profiling the gene expression [15–17]. To achieve the goal of high-quality RNA extraction from plant and animal tissues, various methods have been adopted over the years. However, palmacea consists of species that has hard tissues; therefore, it is very difficult to extract intact RNA in higher amounts, especially from coconuts. Coconuts are mainly produce in the tropical region of world, including China, and Wenchang is famous for its elite coconuts with diverse germplasm. To study the various attribute of the coconut palm at the molecular level, one should be certain of the RNA that can be used effectively in the downstream experiments. The Biotechnology Laboratory at the Coconut Research Institute is actively involved in the extraction of quality RNA from the complex matrices of the coconut species. Quite recently, the laboratory has rigorously tested various methods, such as CTAB, TRIZOL and Tiangen RNA plant for the extraction of RNA from the coconut, yet all the methods failed [7]. Therefore, we have developed a new method (QRREM), which is cost effective, easy and less time consuming. The method has efficiently extracted quality RNA from the matrices that are high in carbohydrates, lipids, proteins and polyphenols. Looking in to the chemical composition and steps of the various methods, it can be concluded that proper combination of chaotropic agent, polyphenol and a polysaccharide binding agent might be necessary in the lysis buffer. Besides, the lipid removing agent will aid in the isolation of quality RNA from the oil rich tissues. The presence of the high amounts of macromolecules (carbohydrates and lipids) in the coconut tissues might have affected the efficiency of the column used for RNA extraction in RNA plant (a commercial kit). Correspondingly, the buffer composition of CTAB and Trizol might lack one or more important component(s) in the recipe that are vital to extract RNA from the coconut tissues.

The QRREM buffer was prepared with all the necessary components, which were required for the successful isolation of RNA from the complex tissues of the coconut (abundant in polysaccharides and lipids with appreciable quantities of polyphenols and proteins). That is the key of the QRREM method to provide with a highly intact RNA in larger amounts with in short time (1.5 h). The current method not only has been validated for the extraction of RNA from coconut tissues, but was expanded for betel nut and the available plant species (mint and spring onion) at the farm of the Coconut Research Institute, Hainan-China. The method might also be useful to extract quality RNA from various animal tissues that are rich in macromolecules and secondary metabolites. In fact, the method will serve the scientific community of the coconut producing areas around the world in order to understand various mechanisms of coconut crop physiology and to develop high yielding resistant cultivars in future.

## Data Availability

All the data are included in the manuscript.

## Abbreviations

CTAB: cetyltrimethylammonium bromide
RT-PCR: reverse transcription real-time PCR
RT-qPCR: quantitative real-time PCR
